# Cancer metabolism and dietary interventions

**DOI:** 10.20892/j.issn.2095-3941.2021.0461

**Published:** 2022-12-22

**Authors:** Lin Qian, Fan Zhang, Miao Yin, Qunying Lei

**Affiliations:** 1 Fudan University Shanghai Cancer Center & Institutes of Biomedical Sciences, Cancer Institutes, Key Laboratory of Breast Cancer in Shanghai, Shanghai Key Laboratory of Radiation Oncology, Shanghai Key Laboratory of Medical Epigenetics, Shanghai Medical College, Fudan University, Shanghai 200030, China; 2 Department of Oncology, Shanghai Medical College, Fudan University, Shanghai 200030, China; 3 State Key Laboratory of Medical Neurobiology, Fudan University, Shanghai 200030, China; 4 Lead Contact, Shanghai 200030, China

**Keywords:** Cancer metabolism, diet intervention, carbohydrate, amino acid, lipid

## Abstract

Metabolic remodeling is a key feature of cancer development. Knowledge of cancer metabolism has greatly expanded since the first observation of abnormal metabolism in cancer cells, the so-called Warburg effect. Malignant cells tend to modify cellular metabolism to favor specialized fermentation over the aerobic respiration usually used by most normal cells. Thus, targeted cancer therapies based on reprogramming nutrient or metabolite metabolism have received substantial attention both conceptually and in clinical practice. In particular, the management of nutrient availability is becoming more attractive in cancer treatment. In this review, we discuss recent findings on tumor metabolism and potential dietary interventions based on the specific characteristics of tumor metabolism. First, we present a comprehensive overview of changes in macronutrient metabolism. Carbohydrates, amino acids, and lipids, are rewired in the cancer microenvironment individually or systematically. Second, we summarize recent progress in cancer interventions applying different types of diets and specific nutrient restrictions in pre-clinical research or clinical trials.

## Introduction

Metabolic remodeling is a key cancer characteristic^1,2^. Research in the past decade has broadened understanding of the critical roles of metabolism in cancer development. Advancements in cancer metabolism have elucidated onco-signaling pathway and metabolic pathway cross-talk, the acquisition of neo- or nonclassical catalysis activity for metabolic enzymes under stress conditions, and the identification of oncometabolites. One notable advancement is the expanded understanding of cancer metabolism beyond original observation, i.e. aerobic glycolysis, known as the Warburg effect, to the rewired utilization of various nutrients, including glucose, amino acids, lipids, and other carbon and/or nitrogen suppliers, such as acetate^3,4^. The abnormal alterations in nutrient metabolism drive uncontrolled cancer cell proliferation and modulate the tumor microenvironment, thereby facilitatingother malignant tumor cell behaviors such as cancer invasion and metastasis^1,2,5^. Therefore, metabolic interventions, particularly the management of nutrient availability, are becoming more attractive for cancer treatment.

## Carbohydrate metabolism in cancer and low-carbohydrate diets in cancer treatment

**Figure 1:**
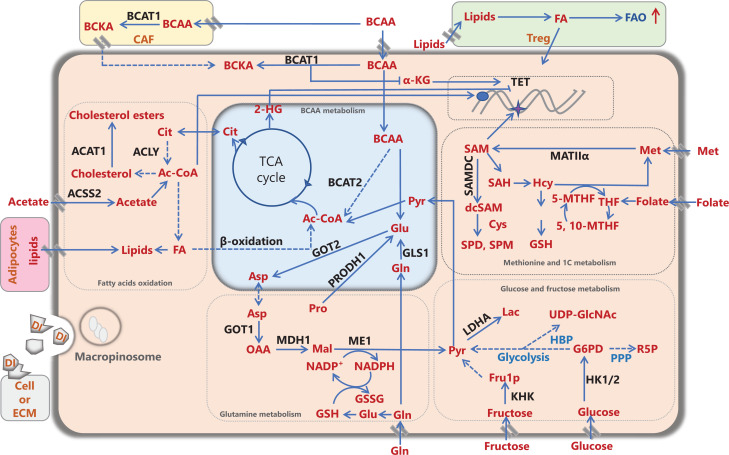
Metabolic remodeling of cancer cells and the cancer microenvironment. Nutrients and/or intermediate metabolites assimilated from the diet or derived from extracellular matrix molecules or stromal cells in the tumor microenvironment, such as carbohydrates, amino acids, and lipids, are involved in rewiring cancer metabolism to meet energy and biomass synthesis requirements, and support cancer development. Ac-CoA: acetyl-CoA, ACLY: ATP citrate lyase, ACAT1: acetyl-CoA acetyltransferase 1, ACSS2: acetyl-CoA synthetase; Ala: alanine, Asp: aspartate, BCAA: branched-chain amino acid, BCAT1/2: branched-chain amino acid transaminase 1/2, BCKA: branched-chain α-ketoacid, CAF: cancer-associated fibroblast, Cit: citrate, Cys: cysteine, DI: degraded ingredient, FA: fatty acid, FAO: fatty acid oxidation, FASN: fatty acid synthase, Fru1P: fructose-1-phosphate, Gln: glutamine, Glu: glutamate, GLS1: glutaminase 1/glutaminase kidney isoform, mitochondrial, GOT1/2: glutamic-oxaloacetic transaminase/aspartate aminotransferase 1/2, G6P: glucose-6-phosphate, GSH: glutathione, GSSG: oxidized glutathione, HBP: hexosamine biosynthetic pathway, Hcy: homocysteine, 2-HG: 2-hydroxyglutarate, HK1/2: hexokinase 1/2, α-KG: α-ketoglutarate, KHK: ketohexokinase, Lac: lactate, LDHA: lactate dehydrogenase A, Mal: malate, ME1: NADP-dependent malic enzyme 1, Met: methionine, MAT IIα: methionine adenosyltransferase IIα, MDH1: malate dehydrogenase 1, NADP^+^: nicotinamide-adenine-dinucleotide phosphate, NADPH: reduced NADP, OAA: oxaloacetate, PPP: pentose phosphate pathway, Pro: proline, PRODH1: proline dehydrogenase, Pyr: pyruvate, R5P: ribose-5-phosphate, SAH: S-adenosylhomocysteine, (dc) SAM: (decarboxylated) S-adenosylmethionine, SAMDC: S-adenosylmethionine decarboxylase, SPD: spermidine, SPM: spermine, TCA: tricarboxylic acid cycle, TET: ten-eleven translocation, THF: tetrahydrofolate, 5-MTHF: 5-methyltetrahydrofolate, 5,10-MTHF: 5,10-methylenetetrahydrofolate, Treg: regulatory T cell, UDP-GlcNAc: UDP-N-acetylglucosamine.

Carbohydrates, including fructose, glucose, and other sugars, are abundant components in sweet fruits and in grains such as wheat, rice, or corn. Carbohydrates serve as the energy source for various life activities. Building blocks produced through enhanced glycolysis have been demonstrated to sustain the uncontrolled proliferation of cancer cells. In addition to well-defined function of glucose in tumor development, recent studies have shown that increased fructose uptake contributes to reinforcing glycolysis and serine metabolism in acute myeloid leukemia (AML) cells^6,7^ (**Figure 1**). In comparison to fructose treatment, which drives glycolysis and promotes colon cancer development in mice, treatment with mannose, another type of monosaccharide, disrupts glycolysis, constrains tumor development, and increases the lethal response to chemotherapy agents^8^. Epidemiologic analysis of the association between sugar uptake and carcinogenesis has indicated that the consumption of foods or beverages with high sugar content is positively correlated with predisposition to cancer^9,10^.

High blood glucose levels are associated with increased incidence of various cancer types, such as pancreatic cancer, endometrial cancer, urinary tract cancer, and malignant melanoma^11^. Diabetes, a constitutive state of high blood glucose, has also been identified as a risk factor for cancer. Insulin is the key factor regulating blood glucose homeostasis. Hyperglycemia leads to increased plasma insulin levels, which are considered the critical cause of tumorigenesis: the activation of insulin and/or insulin-like growth factor-1 (IGF-1) signaling pathways potentiates cell proliferation, survival, and other malignancies^12^. Therefore, an attractive approach involves treating patients with cancer with a low-carbohydrate diet to starve cancer cells and simultaneously normalize plasma insulin levels^12^. Indeed, the ketogenic diet, a typical low-carbohydrate diet, has been developed and applied to cancer treatment. Because of its high fat and low carbohydrate composition, the ketogenic diet provides the body with energy in a manner mainly dependent on ketone utilization. Many studies have demonstrated the beneficial effects of the ketogenic diet on metabolic disorders, such as decreasing body weight and plasma insulin^13,14^. Notably, intractable insulin upregulation has been found to abrogate the therapeutic effects of PI3K inhibitors in clinical trials of targeted cancer therapy. Intriguingly, PI3K inhibitor-stimulated upregulation of plasma insulin level was effectively resolved by administration of PI3K inhibitors together with a ketogenic diet in a mouse tumor model^15^. In addition, the ketogenic diet fundamentally strengthens the immune clearance capability in mice with glioma, thus preventing malignant tumor advancement^16^. Moreover, a clinical study has shown favorable outcomes in patients with cancers, even at late stages, consuming a ketogenic diet^17^.

Blocking the energy supply has long been thought to be an efficient strategy for cancer treatment. In fact, caloric restriction (CR) has been a therapeutic method for tumor growth control for more than a century. CR improves metabolic conditions, such as decreased body weight, blood pressure, plasma insulin, and inflammatory cytokines, and has been shown to have encouraging effects on tumor suppression in various animal models^18^. However, long-term CR remains difficult in humans because of limitations including the lack of tolerance to insufficient long-term dietary intake, and diminished body defense includes immune and physical defenses and tissue recovery in patients with cancer^19–21^. Alternatively, intermittent fasting (IF) has been introduced to mitigate the problems associated with chronic CR. A recent report has indicated that IF confers benefits in mice and patients with hormone receptor-positive tumors treated with hormone therapy, thus prolonging the therapeutic response and avoiding resistance. Further investigations have demonstrated that periodic fasting suppresses the serum concentrations of pleiotropic factors, such as plasma insulin and leptin, thus inactivating signaling pathways driving metabolic reprogramming and cancer promotion^22^. Nevertheless, the effects of IF on tumor suppression have been inconsistent in different mouse models. Discrepancies among these studies may have resulted from the metabolic heterogeneity of cancers or the use of non-standardized clinical study designs^20^. Thus, deciphering the heterogeneity of cancer metabolism and establishing standards for clinical trials are urgently required to ensure reliable application of IF to cancer treatment.

## Amino acid metabolism in cancer and amino acid-restricted diets in cancer treatment

The high glutamine avidity of cancer cells indicates the essential role of amino acids in cancer development. Amino acid oxidation, similarly to glucose catabolism, provides carbon flux to the tricarboxylic acid (TCA) cycle and energy production, maintains redox homeostasis, and supplies precursors for biomass synthesis. Moreover, biosynthesis, particularly nucleotide synthesis, requires nitrogen derived from amino acids.

### Reprogrammed glutamine metabolism in cancer

Rewiring of glutamine metabolism has been widely recognized as a salient feature in various cancers. A recent study using a series of cancer models has revealed that cancer cells consume more glutamine than glucose^23^. Generally, glutamine metabolism gives rise to ammonia. In addition to detoxification in the urea cycle, ammonia can be reused as a nitrogen donor for the synthesis of other amino acids, such as aspartate and proline, thus supporting breast cancer cell proliferation^24^. Furthermore, in pancreatic ductal adenocarcinoma (PDAC) cells, the catalytic process of glutamine-glutamate-α-ketoglutarate (α-KG) is switched to glutamine-aspartate in mitochondria, and this is followed by mitochondrial aspartate release facilitated by mitochondrial uncoupling protein 2 (UCP2)^25,26^. The cytoplasm-relocated aspartate drives the generation of pyruvate and NADPH *via* multiple reactions, thus supporting the viability of PDAC cells, which are vulnerable to ROS damage (**Figure 1**). The glutamine-aspartate-malate metabolic axis in PDAC cells is disrupted by methylation of malate dehydrogenase 1 (MDH1), thus suppressing the production of malate from oxaloacetate^27^. Notably, glutamine metabolism has highly complex effects on the tumor microenvironment. Blocking glutamine metabolism in breast cancer cells ensures the cellular level of NADPH derived from glutamine and stabilizes redox homeostasis in infiltrated immune cells, thus enhancing the anti-tumor effects of immune cells^28^. Therefore, remodeling of glutamine metabolism in cancer is a potential therapeutic target. Clinical trials of the glutaminase inhibitor CB-839 in various cancers are ongoing. Intriguingly, treatment with a combination of a glutaminase inhibitor and the ketogenic diet substantially improves survival in mice with glioblastoma while simultaneously decreasing adverse effects^29^. IF abrogates glutamine-enhanced nucleotide synthesis, thus restoring the response to chemotherapy^30^. Consistently, diets with low glutamine content markedly extend the lifespan of mice with medulloblastoma^31^.

### Reprogrammed branched-chain amino acid metabolism in cancer

Three essential amino acids, valine, leucine, and isoleucine, are categorized as branched-chain amino acids (BCAAs). BCAA degradation is initiated by branched-chain aminotransferase (BCAT). Currently, 2 isoforms of BCAT have been identified: BCAT1, which is mainly present in the cytosol, and BCAT2, which is located in mitochondria. Dysregulation of BCAA metabolism in cancers has been intensively studied in recent years. In PDAC, increased concentrations of serum BCAA resulting from protein breakdown in peripheral tissues have been suggested to be an independent risk factor for PDAC^32–34^. Further examination has revealed that KRAS signaling stabilizes BCAT2 protein levels in PDAC cells, thus potentiating BCAA catabolism, supporting nucleotide synthesis and the TCA cycle, and promoting PDAC development. Notably, the KRAS-BCAA-BACK axis increases pancreatic intraepithelial neoplasia lesion formation at very early stages^35^. In parallel, alterations in BCAA metabolism occur in the tumor microenvironment. TGF-β signaling drives BCAA catabolism by acting on BCAT1 in cancer-associated fibroblasts, thus producing branched-chain α-ketoacid, which fuels PDAC cells^36^. PDAC is also considered to originate from acinar-to-ductal metaplasia. Intriguingly, one study has reported that BCAA-derived acetyl-CoA enhances histone acetylation (**Figure 1**) and enters the sterol isoprenoid anabolic process under the direction of the KRAS-PI3K-AKT-ACLY signaling axis in acinar cells, thereby accelerating acinar-to-ductal metaplasia and PDAC development^37^. Contributions of reprogrammed BCAA metabolism have been reported in other types of cancers. Both chronic and acute myeloid leukemia are dependent on BCAT1-mediated BCAA catabolism^38,39^. Mechanistically, BCAT1 expression is controlled at the post-transcriptional level through the interaction between *BCAT1* mRNA and the RNA-binding protein musashi2 (Msi2). Interestingly, similarly to the effects of 2-hydroxyglutarate (the oncometabolite produced by mutant isocitrate dehydrogenase) in suppressing the DNA demethylase ten-eleven translocation (TET), a decrease in α-KG due to elevated BCAT1 activity inactivates TET and enhances the DNA methylation status in AML stem cells (**Figure 1**). Other studies have shown that BCAA sensing through mTOR signaling promotes myeloid malignancy and hepatocellular carcinoma (HCC)^40,41^. Of note, in contrast to the enhanced BCAA catabolism in PDAC and myeloid leukemia, the suppressed BCAA catabolism in HCC results in the accumulation of intracellular BCAAs, which activate mTOR signaling. These different observations of BCAA catabolism- or BCAA sensing-driven tumorigenesis demonstrate that the functions BCAAs are dependent on the tissue context. Overall, targeting of BCAA metabolism would benefit cancer therapy. Indeed, dietary BCAA restriction has been found to markedly delay PDAC development in a mouse model. The effect is comparable to that achieved by specifically inhibiting BCAT2^35,40^. Nevertheless, BCAA supplementation remains controversial in HCC treatment. In an HCC mouse model, increased BCAA uptake has been found to accelerate cancer malignancy. In contrast, outcomes of patients with HCC have been found to be improved by BCAA administration^40,42^. This discrepancy might be because the HCC mouse model does not precisely mimic human HCC development. An additional concern is that BCAA supplementation has been widely used for muscle strengthening. Amelioration of cachexia, a muscle loss that commonly occurs in advanced stage cancers, requires sufficient intake of protein and amino acids. According to studies on BCAA in cancer, the decision to supplement BCAA in patients with cancer should be determined carefully.

### Reprogrammed methionine and one carbon metabolism in cancer

Methionine metabolism produces a plethora of essential intermediate metabolites. The conversion of methionine to S-adenosyl methionine (SAM) is catalyzed by methionine adenosyltransferase (MAT). Three MAT isoenzymes, I, II, and III, are present in mammalian cells. Whereas MAT I and III are downregulated, MAT II is upregulated at the transcriptional, post-transcriptional, or post-translational levels, and subsequently drives tumorigenesis by accelerating the methionine cycle in various cancers^43–45^. SAM is the major methyl group donor for the methylation of various molecules, such as nucleic acids, proteins, and lipids. Functionally, the epigenetic regulation of histone methylation plays critical roles in the tumor microenvironment (**Figure 1**). Increased uptake of methionine in cancer cells through SLC43A2 transporters impedes methionine consumption by CD8^+^ T cells, thus resulting in decreased intracellular SAM, hypomethylation of histones, and nullification of the anti-tumor effects of CD8^+^ T cells^46^. However, the effects of methionine metabolism on immune cells in cancer have been suggested to have spatial and temporal dependence. One contradictory study has reported elevated SAM and activated methionine salvage pathway in erosion of CD8^+^ T cells in late stage HCC^47^. Further studies might explore whether the opposite effects of SAM on CD8^+^ T cells might be attributable to the function of SAM as a signaling molecule that is sensed by SAMTOR and consequently triggers mTORC1 signaling or other unknown signaling pathways^48^. In cells, metabolic flux from SAM to the polyamine synthesis pathway is controlled by adenosylmethionine decarboxylase, whose overexpression or suppression intriguingly generates malignant transformation of cells through MAPK signaling; thus, both SAM and polyamine might serve as signaling molecules that promote oncogenic effects^49^ (**Figure 1**). S-adenosylhomocysteine and homocysteine are produced sequentially after the removal of the methyl group from SAM. In the presence of inadequate exogenous cysteine in the tumor microenvironment, homocysteine becomes the main source of the antioxidant glutathione through transsulfuration reaction-stimulated cysteine synthesis, thus promoting cancer cell growth and protecting against ferroptosis^50,51^. Finally, homocysteine obtains a methyl group from betaine or 5-methyl-tetrahydrofolate and completes the methionine cycle. Therefore, methionine metabolism is closely associated with folate-mediated one carbon metabolism, which integrates the metabolism of serine, glycine, and methionine; the synthesis of purines and thymidine; methylation; and redox maintenance^52–54^ (**Figure 1**). For example, MAT IIα degradation is triggered by acetylation and/or ubiquitination modifications when folate is absent in hepatocellular and/or colorectal cancer cells^45,55^. On the basis of current evidence, limiting methionine in the diet has been broadly investigated^56,57^. One recent study has reported a rapid decrease in plasma levels of methionine and its intermediate metabolites (within less than 48 h) in response to methionine restriction. However, methionine restriction has minor effects on methionine metabolism in mouse models of various types of cancers. In contrast, the combination of methionine restriction with chemotherapeutic reagents or irradiation therapy significantly increases anti-tumor effects by mitigating methionine metabolism *in vivo* and *in vitro*^56^.

### Reprogrammed serine and glycine metabolism in cancer

Abnormal elevation of serine and glycine synthesis occurs in various cancer cells and contributes to malignant processes^54,58,59^. For example, PDAC cells acquire a growth advantage by using exogenous serine^60^. In contrast, a study on the effect of serine/glycine metabolism on immune cells has revealed that serine/glycine metabolism sustains biosynthesis, and consequently promotes the proliferation of T cells and triggers cytokine production in macrophages^61,62^. Nevertheless, how serine/glycine metabolism educates immune cells in the tumor microenvironment remains elusive. Serine dietary interventions have also been explored. Serine starvation together with inhibition of serine synthesis markedly delays tumor cell growth *in vivo* and *in vitro*^60,62^. Notably, rewired serine metabolism is linked to not only one carbon metabolism but also lipid synthesis in cancers^63^. Sphinganine and deoxysphinganine are lipid metabolites produced from serine palmitoyltransferase-catalyzed metabolism of serine and alanine. Deoxysphinganine is toxic and severely detrimental to the growth of cancer cells cultured in a 3-dimensional matrix. Cancer cell growth is prominently suppressed *in vivo* and *in vitro* under serine/glycine deficiency in the culture medium or in the diet, coupled with serine synthesis inhibition^63^.

### Reprogrammed arginine metabolism in cancer

Arginine metabolism integrates the TCA cycle, the urea cycle, and the synthesis of nitric oxide and polyamine. In addition, arginine acts as a signaling molecule activating the mTORC1 pathway^64–66^. A growing body of evidence demonstrates that arginine-derived nitric oxide, polyamine, and/or arginine activated-signaling substantially promote cancer development^67,68^. Owing to the obstruction of endogenous arginine synthesis caused by a lack of argininosuccinate synthetase 1 (ASS1) and/or ornithine transcarbamylase expression, the arginine supply in many cancer cells depends on exogenous input. Therefore, elimination of cancer cells with arginine auxotrophy might be achieved by depleting cellular arginine. Thus, enzymes including arginine deiminase and human arginase have been introduced to promote arginine degradation and prevent cancer progression^67,69^. Additional evidence has shown that impaired autophagy in a genetic mouse model results in increased plasma ARG1, which removes arginine and alleviates tumor burden^70^. In contrast, a high arginine diet revives tumor growth. Pre-clinical and clinical data from patients with AML show that arginine sufficiency in the tumor microenvironment determines the tumor suppressive effects of T cells. Active T cell responses become moderate when arginine levels are inadequate^71^. Maintenance of serum arginine concentrations by arginase inhibition successfully augments T cell expansion. Thus, in some circumstances, arginine-enriched feeding may benefit T cell therapy in patients with malignancy.

### Reprogrammed proline metabolism in cancer

Proline is synthesized from either ornithine in the cytosol or glutamate in the mitochondria. Both pathways produce a common intermediate metabolite, pyrroline-5-carboxylate (P5C). Conversion of P5C to proline is catalyzed by MYC or PI3K-induced pyrroline-5-carboxylate reductases (PYCRs)^72,73^. Proline synthesis involves reduction reactions and depends on NADP(H) generated by NAD kinase 2 (NADK2) in the mitochondria^74,75^. In the reverse reaction, proline is degraded to P5C by P53, PPARγ, or AMPK-upregulated proline dehydrogenase (PRODH). The catabolism of proline includes oxidation of P5C, thus producing glutamate and giving rise to ROS. Research has clearly established the critical role of proline metabolism in cancer development. PYCR1 expression is upregulated in liver and lung cancers and is associated with poor outcomes in patients. Functionally, increased proline synthesis increases cancer growth, whereas disruption of proline synthesis by inhibition of PYCR1 suppresses tumor growth^76–78^. Further evidence has indicated that Kaposi’s sarcoma development is dependent on the activation of proline synthesis through the interaction between the KI oncoprotein and PYCR1 in tumor cells^79^. In contrast, PRODH-mediated proline degradation contributes to cancer cell invasion and metastasis under certain stress conditions^80^. Proline is ubiquitously present in collagen and other extracellular matrix (ECM) molecules. Cells endocytose degraded cell remnants and ECMs through macropinocytosis (**Figure 1**). ECM-derived proline is a major source in PDAC cells with PRODH overexpression in nutritionally deficient microenvironments^81^. According to the aforementioned studies, altered proline metabolism is expected to be a favorable target for cancer treatment. Indeed, a cluster of cancer cells with inherited defects in endogenous proline synthesis, when cultured in the absence of proline, have been found to show elevated endoplasmic reticulum stress and perturbed cell growth and survival. Moreover, a proline-deficient diet significantly suppresses the growth of xenografted cancer cells in concert with reprogramming of amino acid metabolism, such as a decrease in glutamine, arginine, and the intermediate metabolite ornithine^82^.

### Reprogrammed metabolism of other amino acids in cancer

In addition to the amino acids described above, in recent years, essential roles of other amino acids in cancer development have been reported. Tryptophan in mammals is dependent on dietary assimilation, and its breakdown through the serotonin or kynurenine pathway generates many bioactive metabolites. Kynurenine accumulation is observed in a variety of cancers because of the overexpression of the rate-limiting enzyme, indoleamine 2, 3-dioxygenase 1 (IDO1), and/or tryptophan 2,3-dioxygenase 2 (TDO2). Mechanistic studies have revealed that binding between kynurenine and the transcription factor aryl hydrocarbon receptor initiates signaling that stimulates the amplification of regulatory T cells (Tregs) and the growth of cancer cells, thus leading to an immunosuppressive tumor microenvironment^83–85^. On the basis of these findings, strategies for cancer immunotherapy targeting the kynurenine pathway are being developed. Interestingly, one study has found that tetrahydrobiopterin (BH4) disables the immunosuppressive function of kynurenine and triggers the immune response^86^. Both specific inhibitors of IDO1 and dual inhibitors of IDO and TDO have shown disappointing results in clinical trials to date. Serum kynurenine increases to normal levels or higher after being initially suppressed when inhibitors are used^87,88^. Acquired resistance to inhibitors of IDO1 or IDO1/TDO2 indicates the presence of potential compensatory effects ensuring tryptophan metabolism. Thus, systemically decreasing tryptophan catabolism through nutrition intervention may be promising. Indeed, caloric restriction and/or a ketogenic diet have been found to successfully downregulate the kynurenine pathway^89^.

Although adults can synthesize histidine, daily exogenous supplementation remains necessary. Formimidoyltransferase cyclodeaminase, a dual-function enzyme in histidine catabolism, sequentially produces glutamate and 5,10 methenyl-THF. A recent study has shown that the crosstalk between histidine metabolism and folate cycle enhances the therapeutic response of cancer cells to methotrexate by increasing histidine uptake *in vivo and in vitro*. Nevertheless, the functions of histidine metabolism in cancer development probably depend on the tissue of origin: one study has revealed that *Myc*-driven cancer growth is sustained by histidine supplementation. The leading cause of death in patients with cancer is distant metastasis. Intriguingly, breast cancer metastasis but not growth at the primary site is controlled by asparagine. Inhibition of asparagine synthesis or the removal of asparagine in the diet dramatically impairs metastasis of breast cancer cells^90^.

## Lipid metabolism in cancer and high-fat diets

Lipids play essential roles in various physiological events, such as phospholipid-mediated assembly of cell membranes; steroid or sphingolipid-directed signal transduction; and fatty acid (FA)-balanced energy homeostasis. FA metabolism consists of FA synthesis/lipogenesis and degradation/lipolysis. The initial substrate entering FA synthesis is cytosolic acetyl-CoA (Ac-CoA) derived from citrate or acetate. Conversion of citrate or acetate to Ac-CoA is catalyzed by ATP-citrate lyase (ACLY) or acetyl-CoA synthetase (ACSS), respectively (**Figure 1**). Both ACLY and ACSS1/2 protein levels are elevated in cancer cells, thereby generating Ac-CoA, the synthetic substrate for FA and/or the acetyl-group donor for the acetylation of histone and nonhistone proteins^91–93^. Lipolysis and FA oxidation (FAO) break down lipids, thus yielding Ac-CoA, which enables the TCA cycle when glucose is insufficient to generate pyruvate (**Figure 1**). Cancer cells frequently encounter energy crises due to hypoxia and vascular dysfunction. To overcome these obstacles, cells can hijack FAO to satisfy energy demands. Carboxylesterase 1 (CES1)/triacylglycerol hydrolase is upregulated in obese patients with colorectal cancer, thus guiding cancer development by reinforcing triacylglycerol degradation and FAO^94^. Hijacking FAO also helps AML stem cells develop resistance to venetoclax^95^.

The association between cholesterol metabolism and cancer development has long remained elusive. Nevertheless, with multi-omics tools, several studies have uncovered the reshaping of cholesterol metabolism in cancer cells. Analysis of data from The Cancer Genome Atlas (TCGA) has revealed that oncogenic signaling stimulates cholesterol synthesis and translocalization^96^. An additional study using proteomics data has demonstrated that elevated protein levels of sterol O-acyltransferase 1 (SOAT1)/acyl-coenzyme A:cholesterol acyltransferase 1 (ACAT1), which catalyzes cholesterol esterification, substantially promote malignant phenotypes of HCC cells *in vivo* and *in* *vitro*^97^ (**Figure 1**).

Emerging evidence in recent years has revealed that lipid metabolism primes the cancer microenvironment and facilitates cancer development. Adipocytes, the main lipid depot in the body, engage in intensive crosstalk with cancer cells through paracrine or endocrine pathways. Adipocyte-released factors, such as hormones (leptin), ECM molecules (collagen VI and endotrophin), and metabolites (glutamine and creatine), contribute to the growth, metastasis, and chemo-resistance of cancer cells^98–101^. In particular, adipocytes in the microenvironment directly feed lipids to ovarian cancer cells, thus supporting cancer growth^102^. In recent decades, research on lipid metabolism disorders in the tumor immune microenvironment have become a focus to refine cancer immunotherapy. In the tumor microenvironment, neutrophils with increased lipogenesis display behaviors similar to those of adipocytes, by transferring lipids to metastatic breast cancer cells and providing energy^103^. Intriguingly, compared with its promoting effect on cancer cell growth, cholesterol esterification constrains CD8^+^ T cell proliferation and cytotoxic effects^104^. In contrast, Tregs isolated from cancers obtain more exogenous lipids by enhancing CD36-mediated transport, thus resulting in immunosuppressive functions^105^. Another study has revealed that FA synthesis promotes Treg maturation^106^ (**Figure 1**). Furthermore, immunosuppression of the tumor microenvironment may be attributed to impaired NK cell function due to FAO-triggered ROS overloading^107^.

In line with the aberrant lipid metabolism in neoplastic and/or cancer cells, epidemiological evidence has confirmed a positive correlation between obesity and various types of cancers^108,109^. To investigate a potential causative relationship, researchers have used mouse models with tumor xenografts or activated oncogenic signaling together with high-fat diet (HFD) or fat-enriched diet feeding. These diets differ from the ketogenic diet and are characterized by high fat and low carbohydrate content. HFDs profoundly potentiates malignant progress in pancreatic, liver, prostate, colon, and breast cancers^110–114^. Moreover, HFDs synergistically induce the malignant transformation of liver cells or elicit tumorigenesis by nourishing stem cells or suppressing the tumor immune microenvironment^115–117^.

## Conclusions

The metabolism of nutrients such as carbohydrates, amino acids, and lipids is rewired individually or systematically in cancers according to the disease stage, the surrounding microenvironment, and/or tissue of origin, thus resulting in metabolic heterogeneity and increasing the chance of resistance to monotherapies. Notably, metabolic alterations occurring at pre-cancerous lesions in response to nutritional stress or oncogenic signaling can drive tumorigenesis^35,118,119^. Therefore, a tailored diet based on the metabolic specificity in individual patients would greatly facilitate cancer prevention and treatment in patients with oncogenic mutations, pre-cancerous lesions, or cancers by arresting malignant transformation, increasing sensitivity to immunotherapy, decreasing drug resistance, and ameliorating adverse effects. Finally, gut microbes produce large amounts of metabolites by interacting with various components of the diet and affecting the absorption of digested molecules, thus globally regulating metabolism in the body. The disruption of gut microbe metabolic homeostasis increases the susceptibility to certain cancers^120,121^. Deciphering the interactions between gut microbe metabolism and the diet should pave the way to the development of novel therapies against cancer.
